# Discovery and Identification of Four Novel Species of *Distoseptispora* (Distoseptisporaceae, Distoseptisporales) on Decaying Wood from Hainan and Fujian Provinces, China

**DOI:** 10.3390/jof11090667

**Published:** 2025-09-11

**Authors:** Wenwen Liu, Changzhun Yin, Yang Jiang, Xigang Yan, Xingsheng Wang, Xiuguo Zhang, Shi Wang

**Affiliations:** 1 College of Life Sciences, Shandong Normal University, Jinan 250358, China; 17615593869@163.com (W.L.); zcy94156@163.com (C.Y.); jiangyang202309@126.com (Y.J.); yxg210911@126.com (X.Y.); 18560178820@163.com (X.W.); zhxg@sdau.edu.cn (X.Z.); 2 Shandong Provincial Key Laboratory for Biology of Vegetable Diseases and Insect Pests, College of Plant Protection, Shandong Agricultural University, Taian 271017, China

**Keywords:** dematiaceous hyphomycetes, morphology, new species, phylogenetic, Sordariomycetes, taxonomy

## Abstract

Decaying wood, as a unique substrate, which contains cellulose, hemicellulose, lignin, and nitrogen-containing compounds, harbors significant fungal biodiversity, particularly among dematiaceous hyphomycete species. This study uses a combination of morphological and molecular systematics methods. Phylogenetic analyses of ITS, LSU, *RPB2* and *TEF1* sequences were conducted using the maximum likelihood (ML) and the Bayesian inference (BI) method. Combined with the morphological characteristics, four new species of *Distoseptispora*, *D. bawanglingensis* sp. nov., *D. changjiangensis* sp. nov., *D. daanyuanensis* sp. nov. and *D. jianfenglingensis* sp. nov., were identified from decaying wood collected in Hainan and Fujian provinces, China. This study provides detailed descriptions, illustrations, and phylogenetic trees with the aim of clarifying the taxonomic status of these four new species, thereby enhancing our understanding of the species diversity of *Distoseptispora* in Hainan and Fujian provinces, China.

## 1. Introduction

*Distoseptispora* K.D. Hyde, McKenzie & Maharachch., a dematiaceous hyphomycete, has been reported and extensively studied in recent years. Su et al. initially described *Distoseptispora* species, yet subsequent phylogenetic studies revealed its lack of evolutionary relationship with *Miyoshiella*, thereby necessitating the establishment of this novel genus [1]. Su et al. designated *D. fluminicola* as the type species [1]. At present, the Index Fungorum (http://www.indexfungorum.org/, accessed on 15 May 2025) has a total of 94 epithet entries pertaining to *Distoseptispora*. *Distoseptispora* belongs to the Distoseptisporaceae, Distoseptisporales, Sordariomycetes [2]. Most descriptions of *Distoseptispora* are based on its asexual morphological characteristics, including conidiophores, conidiogenous cells, and conidia, with further differentiation from closely related species [1,3,4,5,6,7,8,9,10]. The asexual morph of *Distoseptispora* is characterized by conidiophores that are typically solitary or clustered, erect or slightly curved, septate, and pale-colored, with conidia that are elongate-ovoid, cylindrical, or fusiform, commonly bearing multiple transverse septa, displaying a smooth or finely textured surface, predominantly light brown or hyaline, and borne terminally or laterally on the conidiogenous cells [1,3,6,8,9]. However, the sasexual morph of *Distoseptispora* has been described by [2,10,11]. Thus, further investigation is required to characterize additional sasexual morphs within the genus *Distoseptispora*.

Due to prolonged exposure to a humid environment, the wood began to decay and gradually lost its structural integrity and mechanical strength, which provided a favorable environment for the growth of fungi. *Distoseptispora* is principally parasitic on decaying wood and other plant debris, playing a pivotal role in the decomposition of organic matter. Tibpromma et al. isolated and identified two novel species, *Distoseptispora thailandica* and *D. xishuangbannaensis*, from decaying leaves of *Pandanus* sp. and *Pandanus utilis*, respectively, and updated the phylogenetic tree for the family Distoseptisporaceae [8]. Monkai et al. described *Distoseptispora hydei*, which had been isolated from decaying bamboo culm in Phitsanulok Province, Thailand, this species, *D. hydei*, exhibits distinctive conidial features, namely obpyriform to fusiform conidia bearing 7–9-distoseptate and being encased in a hyaline, gelatinous sheath at the apical region3 [11]. In addition, Sun et al. further expanded the genus by reporting *Distoseptispora bambusae*, from bamboo culms in China and Thailand [7]. Jayawardena et al. isolated and identified *Distoseptispora bambusicola* from decaying wood of bamboo in freshwater in Zunyi, Guizhou [6]. Subsequently, morphological comparisons and phylogenetic analyses were conducted between *D. bambusicola*, *D. hydei*, *D. obpyriformis*, and *D. rostrata*. Konta et al. reported *Distoseptispora licualae* from dead leaves of *Licuala glabra*, and provided descriptions and illustrations of its sexual morph [12]. Hyde et al. reported the occurrence of *D. phragmiticola* on *Phragmites australis*, thereby further enriching the host diversity of the genus *Distoseptispora* [13]. Karimi et al. reported three new species, namely *Distoseptispora arecacearum*, *D. eleiodoxae*, and *D. narathiwatensis*, which parasitize, respectively, on the submerged rachis of *Licuala paludosa*, *Eleiodoxa conferta*, and *Eugeissona tristis* in peat swamp forests [14]. Sun et al. reported six new species of *Distoseptispora* from two host plants [2]. Among these, *Distoseptispora bambusae*, *D. effusa*, *D. gelatinosa*, *D. tectonigena*, and *D. yongxiuensis* were parasitic on bamboo culms in terrestrial habitats, while *D. olivaceoviridis* was parasitic on twigs of *Clerodendrum quadriloculare*. Therefore, we have concluded that a considerable number of *Distoseptispora* species parasitic on decaying wood, particularly bamboo culms. Furthermore, we can conduct more targeted research based on the hosts of *Distoseptispora* to discover more new species of this genus and enrich its species diversity.

The fungal resources on decaying wood substrates are abundant, yet a large number remain to be developed and studied. Therefore, in this study, we aimed to investigate the fungal species inhabiting decaying wood in Hainan Province and Fujian Province based on morphological and phylogenetic analyses. Using morphological characteristics and multi-gene phylogenetic analysis (ITS, LSU, *RPB2* and *TEF1*), we isolated and identified four new species of *Distoseptispora*. Additionally, we discussed and compared the differences between these new species and their closely related species in Table 1 thereby contributing to the enrichment of the species diversity within this genus.

## 2. Materials and Methods

### 2.1. Sample Collection and Treatment

In the present study, decaying wood specimens were collected from Hainan Province and Fujian Province, China. Information regarding the collector, collection sites, collection time, altitude, longitude, latitude, habitat, climatic conditions, host plant, and substrate was recorded. Cut the collected decaying wood into segments of an appropriate length. Branch segments were placed in a Petri dish lined with filter paper, followed by the addition of an appropriate volume of sterile water, and incubated in a humidified environment for 2–3 weeks. Simultaneously, decaying wood samples were regularly observed, and sterile water was added to the culture substrate to maintain humidity. Sterile picking needles were used to aseptically harvest conidia and inoculate them onto PDA medium (PDA: 14 g agar, 20 g dextrose, 200 g potato, 1 L distilled water, pH 7.0). Select 3–5 spores from PDA medium and subject them to alternating light and dark culture in a biological incubator maintained at 27 °C. Upon the development of individual colonies, use a sterilized picking needle to aseptically isolate the individual colonies from the culture medium and transfer them onto a fresh PDA medium plate for separation and purification, thereby obtaining pure strains. The entire experimental process requires strict adherence to aseptic techniques.

### 2.2. Morphological and Cultural Characterization

Colonies were cultured for 7, 14, and 21 days, and images of the front and back surfaces were captured using a digital camera (Canon PowerShot G7X; Canon Inc., Beijing, China). Morphological characteristics of the fungi were observed and examined under a stereo microscope (Olympus SZX10; Olympus Optical Co., Ltd., Beijing, China) and a microscope (Olympus BX53; Olympus Optical Co., Ltd., Japan). Structures including conidiophores, conidiogenous cells, and conidia of the fungi were photographed using a high-definition digital camera (Olympus DP80; Olympus Optical Co., Ltd., Tokyo, Japan). Microstructural measurements were conducted using Digimizer software. Typically, 20–30 samples were measured. All strains were preserved in sterile 10% glycerol at 4 °C. Voucher specimens have been deposited in the Herbarium of the Department of Plant Pathology, Shandong Agricultural University (HSAUP), Taian, China, and the Herbarium Mycologicum Academiae Sinicae, Institute of Microbiology, Chinese Academy of Sciences (HMAS), Beijing, China. Ex-type living cultures are deposited in the Shandong Agricultural University Culture Collection (SAUCC). The taxonomic information and morphological description of the new species have been uploaded to MycoBank (http://www.mycobank.org, accessed on 15 May 2025).

### 2.3. DNA Extraction, PCR Amplification, and Sequencing

Fungal DNA was extracted from fungal cultures that had been grown on PDA, utilizing CTAB (cetyltrimethylammonium bromide) [15,16]. Upon achieving adequate mycelial development in the PDA medium, aseptically transfer approximately 0.2 g of fungal biomass into a 1.5 mL microcentrifuge tube using a sterilized scalpel. Introduce CTAB-based lysis buffer to the sample, followed by mechanical disruption of the mycelial matrix via bead-beating homogenization. Subsequent to thorough tissue lysis, incubate the lysate-containing tube in a thermostatically controlled water bath maintained at 65 °C for a duration of 2 h to facilitate complete cell membrane permeabilization and nucleic acid release. Following mechanical disruption of the mycelial sample, the lysate is subjected to centrifugation at 12,000× *g* for 15 min at 4 °C to isolate the aqueous phase. The clarified supernatant is transferred to a sterile tube, followed by the addition of an equal volume of chloroform: isoamyl alcohol (24:1) to induce phase separation and enable selective partitioning of genomic DNA into the aqueous layer. After thorough vortex mixing, a second centrifugation step is performed. The upper aqueous layer is subsequently collected, and the chloroform: isoamyl alcohol extraction is repeated to enhance DNA purity prior to precipitation [17,18]. Polymerase Chain Reaction (PCR) amplification of extracted fungal genomic DNA was performed using ITS, LSU, *RPB2*, and *TEF1* in Table 2. The primer pairs used for these genes were as follows: ITS: ITS5/ITS4 [19], LSU: LR0R/LR5 [20,21], *RPB2*: RPB2–5F2 [22]/fRPB2–7cR [23], and *TEF1*: EF1–983F/EF1–2218R [24,25]. PCR amplification was performed in 25 μL reactions containing 9.5 μL ddH2O, 12.5 μL 2× Taq Plus Master Mix (Shanghai, China) (with dye) (Yeasn Biotechnology, Shanghai, China, Cat No. 10154ES03), and 1 μL each of forward/reverse primers (10 μm). Amplicons were resolved by 2% agarose gel electrophoresis stained with ethidium bromide and visualized directly under UV transillumination, with fluorescent bands confirming DNA amplification products [26]. PCR conditions are derived from the reference literature. The synthesis of PCR primers and subsequent DNA sequencing services were contractually outsourced to Tsingke Biotechnology Co., Ltd. (Qingdao, China). Following completion of sequencing runs, bioinformatics analysis was performed using MAGE7 [27] software for multiple sequence alignment and contiguous sequence assembly of the raw sequencing data. The nucleotide sequences of the four novel taxa were deposited in the NCBI GenBank database. Relevant taxonomic literature was retrieved via a targeted search of Index Fungorum (https://indexfungorum.org, accessed 15 May 2025) using the genus name *Distoseptispora* and taxonomic filters to ensure nomenclatural currency. The GenBank accessions cited in this study were compiled and summarized in Supplementary Materials.

### 2.4. Phylogenetic Analysis

Nucleic acid sequences for *Distoseptispora* were retrieved from the National Center for Biotechnology Information (NCBI) database (https://www.ncbi.nlm.nih.gov/, accessed 15 May 2025), and corresponding GenBank accession numbers were extracted from the most recent version of the associated manuscript [28]. Multiple sequence alignment of nucleotide sequences from the four novel taxa and publicly available reference sequences was conducted using MAFFT 7 (http://mafft.cbrc.jp/alignment/server/, accessed 16 May 2025) [29]. Phylogenetic analyses of the aligned sequences were performed using maximum likelihood (ML) and Bayesian inference (BI) algorithms. Both analytical approaches were executed independently through registering on the CIPRES website [30]. Maximum likelihood phylogenetic inference was conducted using RAxML-HPC2 v.8.2.12 on XSEDE resources, employing the GTRGAMMA nucleotide substitution model with 1000 rapid bootstrap replicates for confidence assessment [31]. MrModeltest v.2.3 [32] software was utilized to screen for optimal evolutionary models, while BI was conducted using MrBayes 3.2.7a (on XSEDE) [33,34,35]. The inferred phylogenetic tree was visualized and re-rooted using an outgroup with FigTree v1.4.3 (http://tree.bio.ed.ac.uk/software/figtree/, accessed on 16 May 2025). The final phylogenetic tree was prepared using Adobe Illustrator CC 2019. The final phylogenetic tree displays new species designations highlighted in red for visual differentiation.

## 3. Results

### 3.1. Phylogenetic Analysis

**Figure 1:**
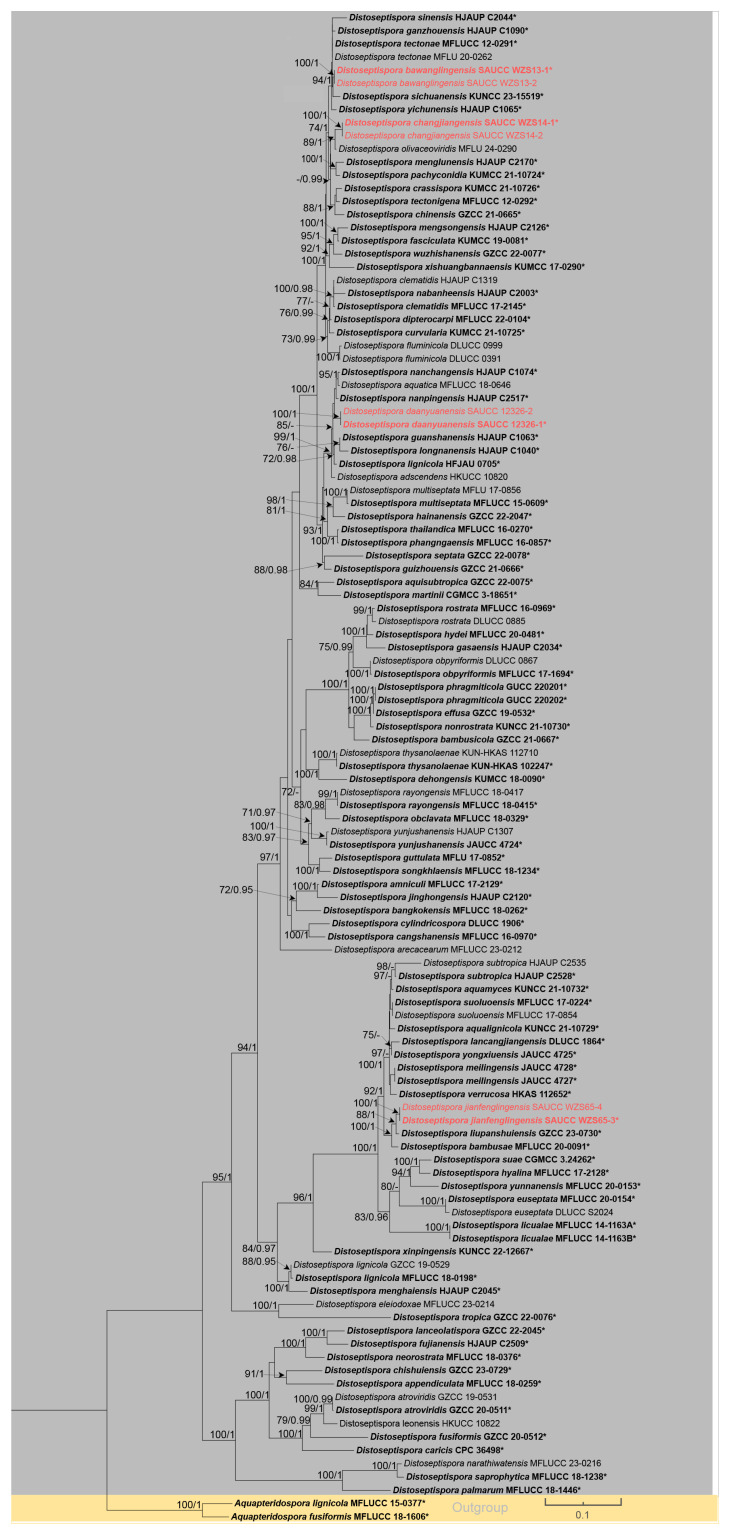
Phylogenetic tree of *Distoseptispora* based on combined ITS, LSU, *RPB2* and *TEF1* sequences. The maximum likelihood (ML) and Bayesian inference (BI) methods bootstrap support values above 70% and 0.90 are separated by MLBS/BPP and new species are highlighted in red. Ex-type or ex-epitype strains are shown in bold and marked with an asterisk ("*"). Branches separated by yellow and gray indicate different species of *Aquapteridospora* and *Distoseptispora*. The lower right quadrant of the diagram illustrates a nucleotide substitution rate of 0.1 substitutions per site, as depicted by the annotated scale.

Evolutionary relationships among *Distoseptispora* taxa were clarified through molecular phylogenetics. Multi-source sequence alignment incorporated GenBank-derived data and newly acquired sequences of new species, utilizing *Aquapteridospora fusiformis* Z.L. Luo, D.F. Bao, Hong Y. Su & K.D. Hyde MFLUCC 18-1606 and *A. lignicola* MFLUCC 15-0377 as phylogenetic outgroups. A combined sequence matrix (n = 115, 2687 bp) was assembled, integrating ITS (1–506), LSU (507–1281), *RPB2* (1282–1872) and *TEF1* (1873–2687) regions for downstream phylogenomic inference. The dataset comprised 1487 constant characters, 259 variable but parsimony non-informative, and 941 parsimony informative characters. The resulting phylogenies from maximum likelihood (ML) and Bayesian inference (BI) analyses show strong topological agreement. Nucleotide substitution models were selected via the Akaike Information Criterion (AIC) using jModelTest2 [36] on XSEDE within the CIPRES web portal [37]. The GTR+I+G model was selected for ITS, the TIM2+I+G model for LSU, and the GTR+I+G model for *RPB2* and *TEF1*. Figure 1 shows the best-scoring maximum likelihood (ML) evolutionary tree, where maximum likelihood bootstrap analyses and Bayesian posterior probabilities (MLBS/BPP) are labeled at node positions. Eight novel *Distoseptispora* isolates were integrated into the phylogenomic analysis, with results visualized in the maximum likelihood (ML) tree. The eight novel isolates characterized herein formed four distinct monophyletic clades in the phylogeny, corresponding to four new *Distoseptispora* species: *D. bawanglingensis*, *D. changjiangensis*, *D. daanyuanensis* and *D. jianfenglingensis.*

### 3.2. Taxonomy

**Figure 2:**
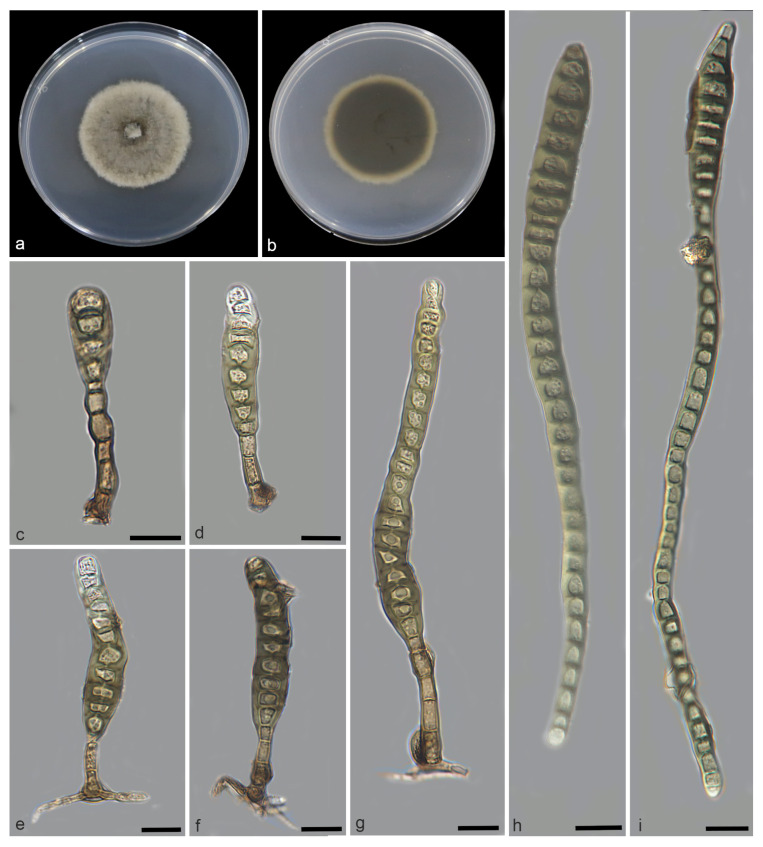
*Distoseptispora bawanglingensis* (holotype: HMAS 354057). (**a**) surface of colony after 3 weeks on PDA; (**b**) reverse of colony after 3 weeks on PDA; (**c**–**g**) conidiophores, conidiogenous cells and conidia; (**h**,**i**) conidia. Scale bars: (**c**–**i**) 10 μm.

**Figure 3:**
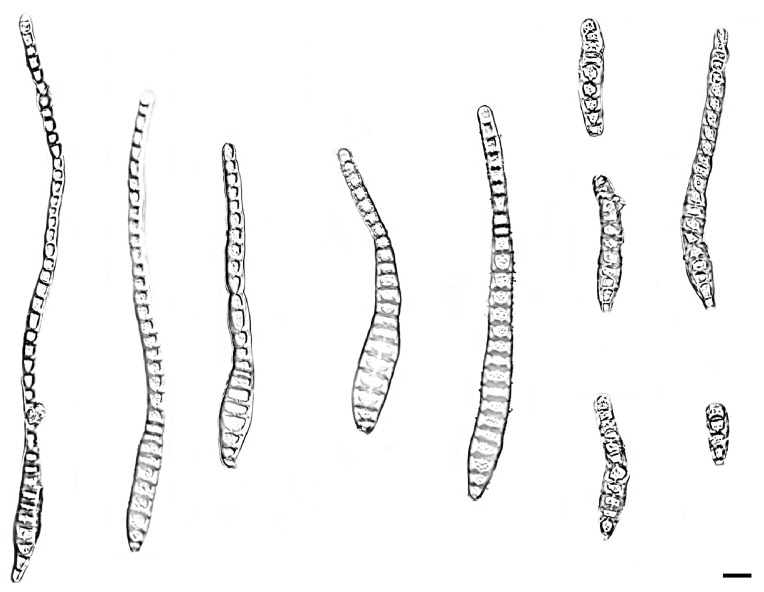
*Distoseptispora bawanglingensis* (holotype: HMAS 354057). conidia. Scale bars: 10 μm.

*Distoseptispora bawanglingensis* W.W. Liu, C.Z. Yin, X.G. Zhang & S. Wang, sp. nov. Figure 2 and Figure 3

MycoBank—MB859425

Holotype—China, Hainan Province, Changjiang Li Autonomous County, Bawangling National Forest Park (109°7′22″ N, 19°5′9″ E), from decaying wood, 14 October 2023, W.W. Liu, holotype HMAS 354057, ex-holotype living culture SAUCC WZS13-1.

Etymology—The epithet “*bawanglingensis*” denotes the collection site of the strains, namely, Bawangling National Forest Park.

Description—Saprobic on decaying wood in peat swamp forest. Sexual morph: Undetermined. Asexual morph: Hyphomycetous. Mycelium immersed to substratum, composed of pale brown, branched, septate, smooth. Conidiophores macronematous, unibranchiate, cylindrical, upright, unbranched, straight to slightly flexuous, smooth or micro-verrucose, single, dark brown, thick-walled, 2–8-septate with lobed basal cells, enteroblastic percurrent extensions, 13.9–40.6 × 3.5–6.7 μm ($\bar{x}$ = 22.5 × 5.2 μm, SD = 9.5 × 0.9, n = 20). Conidiogenous cells terminal, integrated, monoblastic, brown, smooth, cylindrical, 2.5–8.1 × 3.2–6.3 μm ($\bar{x}$ = 5.6 × 3.9 μm, SD = 1.8 × 0.8, n = 20). Conidia acrogenous or obclavate, solitary, straight or slightly curved, brown to pale brown, 3–45-distoseptate, swollen at the proximal end, rostrate, tapering and paler toward the rounded apex, 20.2–206.7 × 8.3–13.7 μm ($\bar{x}$ = 141.7 × 10.4 μm, SD = 72.4 × 1.4, n = 25).

Culture characteristics—After 21 days of dark cultivation at 25 °C on PDA, the colony diameter reached 45 mm, and a growth rate of 1.9–2.3 mm/day. Colonies raised, circular, velutinous edge, grayish-brown, pale edge, surface velvety, felted, dense, reverse concentric, dark brown inner ring, outer pale brown halo.

Additional material studied—China, Hainan Province, Changjiang Li Autonomous County, Bawangling National Forest Par, from decaying wood, 14 October 2023, W.W. Liu, living culture SAUCC WZS13-2.

Notes—Phylogenetic analysis of ITS, LSU, *RPB2*, and *TEF1* sequences revealed that *Distoseptispora bawanglingensis* exhibits a close phylogenetic relationship with *D. sichuanensis*. The nucleotide differences in the *TEF1* and LSU regions between *Distoseptispora sichuanensis* and *D. bawanglingensis* are 8/815 (99% similarity) and 13/775 (98.3% similarity), respectively. Morphologically, there are differences in size and the number of distosepta between the two types of conidia. Specifically, the conidia of *Distoseptispora bawanglingensis* are longer and finer in average size (*D. bawanglingensis*: 20.2–206.7 × 8.3–13.7 μm; $\bar{x}$ = 141.7 × 10.4 μm, n = 25 vs. *D. sichuanensis*: 80–145 × 6–17 μm; $\bar{x}$ = 114 × 12.8 μm, n = 20) [38]; *D. bawanglingensis* has conidia with 3–45-distoseptate, whereas *D. sichuanensis* is characterized by 12–20-distoseptate [39]. Consequently, *D. bawanglingensis* is recognized as a novel species within the genus *Distoseptispora*, supported by phylogenomic analyses and morphological characterizations.

**Figure 4:**
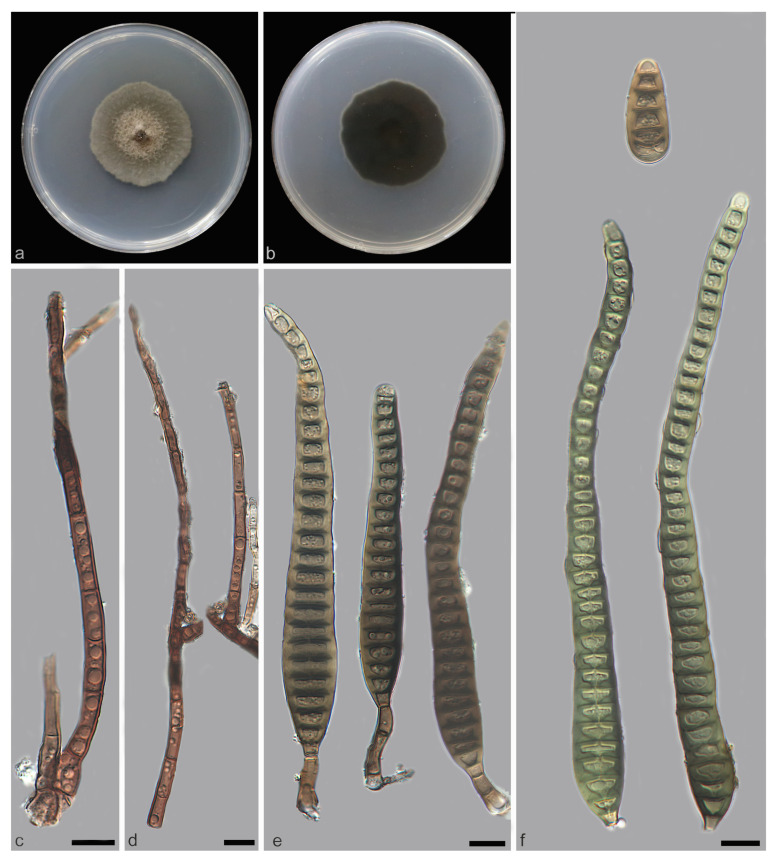
*Distoseptispora changjiangensis* (holotype: HMAS 354058). (**a**) surface of colony after 3 weeks on PDA; (**b**) reverse of colony after 3 weeks on PDA; (**c**,**d**) conidiophores; (**e**) conidiophores, conidiogenous cells and conidia; (**f**) conidia. Scale bars: (**c**–**f**) 10 μm.

**Figure 5:**
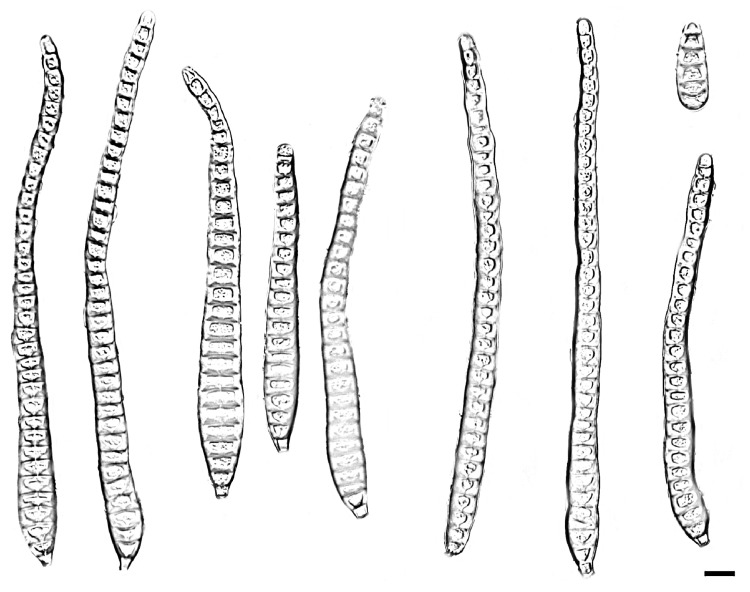
*Distoseptispora changjiangensis* (holotype: HMAS 354058). conidia. Scale bars: 10 μm.

*Distoseptispora changjiangensis* W.W. Liu, C.Z. Yin, X.G. Zhang & S. Wang, sp. nov. Figure 4 and Figure 5

MycoBank—MB859426

Holotype—China, Hainan Province, Changjiang Li Autonomous County, Bawangling National Forest Park (109°7′22″ N, 19°5′9″ E), from decaying wood, 14 October 2023, W.W. Liu, holotype HMAS 354058, ex-holotype living culture SAUCC WZS14-1.

Etymology—The epithet “*changjiangensis*” denotes the collection site of the strains, namely, Changjiang Li Autonomous County.

Description—Saprobic on decaying wood in peat swamp forest. Sexual morph: Undetermined. Asexual morph: Hyphomycetous. Mycelium immersed to substratum, pale brown to subhyaline, branched, septate, smooth. Conidiophores branched or unibranched, cylindrical or rod-shaped, upright, straight to slightly flexuous, smooth or micro-verrucose, dark brown, single, thick-walled, 2–18-septate with lobed basal cells, enteroblastic percurrent extensions, 12.0–173.5 × 4.3–6.5 μm ($\bar{x}$ = 132.1 × 5.1 μm, SD = 52.3 × 0.3, n = 20). Conidiogenous cells terminal, brown, integrated, monoblastic, cylindrical, smooth, 2.3–7.2 × 3.0–6.1 μm ($\bar{x}$ = 3.5 × 5.2 μm, SD = 1.9 × 0.6, n = 20). Conidia acrogenous or obclavate, solitary, straight or slightly curved, brown to pale brown, 6–40-distoseptate, rostrate, swollen at the proximal end, tapering and paler toward the rounded apex, 27.2–160.8 × 12.1–15.8 μm ($\bar{x}$ = 153.6 × 14.1 μm, SD = 49.9 × 0.4, n = 25).

Culture characteristics—After 21 days of dark cultivation at 25 °C on PDA, the colony diameter reached 45 mm, and a growth rate of is 1.9–2.3 mm/day. Colonies circular, velutinous edge, low convex, concentric, light gray inner ring, outer dark gray, grayish-brown at the central parts, surface felted, dense, reverse dark brown, outer grayish white halo.

Additional material studied—China, Hainan Province, Changjiang Li Autonomous County, Bawangling National Forest Par, from decaying wood, 14 October 2023, W.W. Liu, living culture SAUCC WZS14-2.

Notes—Phylogenetic analysis of ITS, LSU, *RPB2* and *TEF1* sequences revealed that *Distoseptispora changjiangensis* formed a well-supported monophyletic group with *D. olivaceoviridis*, indicating their close evolutionary relationship. Morphologically, differences exist in the conidiophores and conidia of the two *Distoseptispora* species. Specifically, the conidiophores of *Distoseptispora changjiangensis* are branched or unbranched with rounded protrusions on the surface, whereas those of *D. olivaceoviridis* are unbranched and lack such rounded protrusions. The conidia of *Distoseptispora changjiangensis* are longer and thicker on average (27.2–160.8 × 12.1–15.8 μm; $\bar{x}$ = 153.6 × 14.1 μm, n = 25) vs. *D. sichuanensis*: (46–115 × 8–12 μm; $\bar{x}$ = 80 × 9 μm, n = 15). Additionally, *D. changjiangensis* has conidia with 6–40-distoseptate, whereas *D. olivaceoviridis* is characterized by 12–16-distoseptate [2]. Consequently, *D. changjiangensis* is recognized as a novel species within the genus *Distoseptispora*, supported by phylogenomic analyses and morphological characterizations.

**Figure 6:**
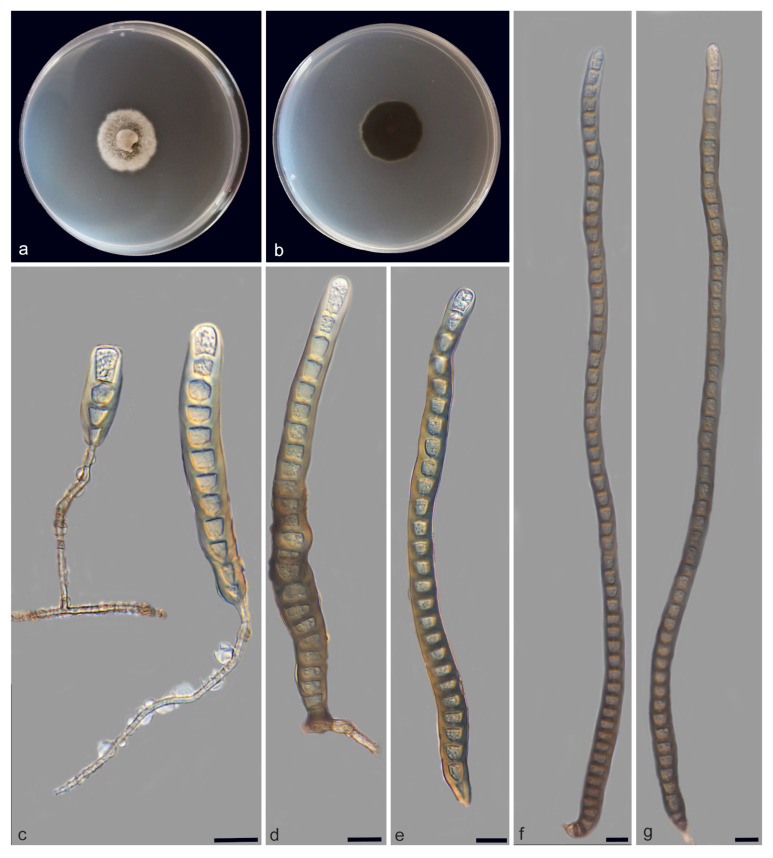
*Distoseptispora daanyuanensis* (holotype: HMAS 354060). (**a**,**b**) colony front and back after 21 days culture on PDA; (**c**) conidiophores, conidiogenous cells and conidia; (**d**) conidiogenous cells and conidia; (**e**–**g**) conidia. Scale bars: (**c**–**g**) 10 μm.

**Figure 7:**
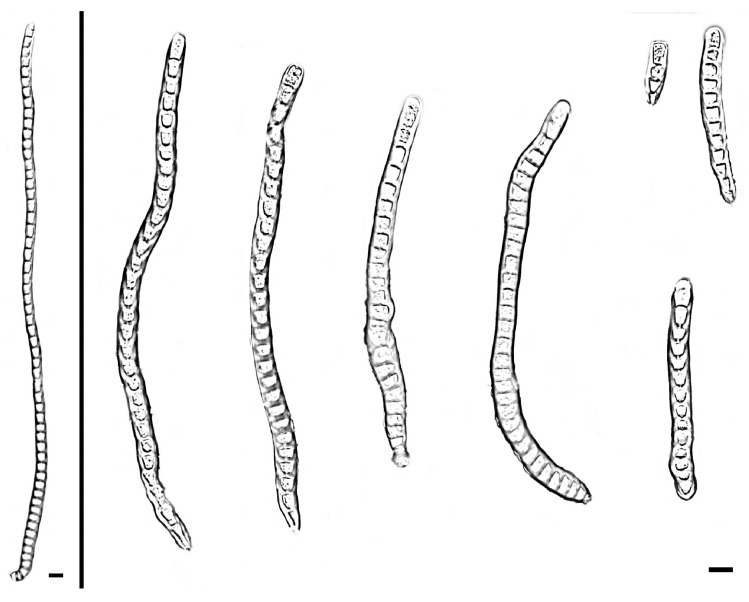
*Distoseptispora daanyuanensis* (holotype: HMAS 354060). conidia. Scale bars: 10 μm.

*Distoseptispora daanyuanensis* W.W. Liu, C.Z. Yin, X.G. Zhang & S. Wang, sp. nov. Figure 6 and Figure 7

MycoBank: MB859427

Holotype—China, Fujian Province, Wuyishan City, Da’anyuan Ecological Tourism Zone (117°57′22″ N, 27°52′25″ E), from decaying wood, 20 October 2024, W.W. Liu, holotype HMAS 354060, ex-holotype living culture SAUCC12326-1.

Etymology—The epithet “*daanyuanensis*” denotes the collection site of the strains, namely, Da’anyuan Ecological Tourism Zone.

Description—Saprobic on decaying wood in peat swamp forest. Sexual morph: Undetermined. Asexual morph: Hyphomycetous. Mycelium embedded within the growth substratum, aerial mycelia medium, branched, smooth, septate. Conidiophores fine, mycelial, mycelium-like, straight to slightly flexuous, verrucose, branched, brown to white, and closely related to the mycelial structure, 25.5–46.7 × 1.3–2.4 μm ($\bar{x}$ = 38.4 × 1.8 μm, SD = 12 × 0.7, n = 20). Conidiogenous cells terminal, light brown, holoblastic, cylindrical, integrated, monoblastic, smooth. Conidia acrogenous, obclavate, solitary, straight or slightly curved, brown to pale brown, 4–92-distoseptate, rostrate, swollen at the proximal end, tapering and paler toward the rounded apex, 23.1–696.2 × 9.3–17.9 μm ($\bar{x}$ = 302.4 × 11.3 μm, SD = 212.8 × 3.1, n = 25).

Culture characteristics—After 21 days of dark cultivation at 25 °C on PDA, the colony diameter reached 25 mm, and a growth rate of is 0.9–1.6 mm/day. Colonies circular, white, with gray exudates, velutinous edge, dull surface, felted, dense, reverse concentric, blackish-brown inner ring, outer grayish white halo.

Additional material studied—China, Fujian Province, Wuyishan City, Da’anyuan Ecological Tourism Zone, from decaying wood, 20 October 2024, W.W. Liu, living culture SAUCC12326-2.

Notes—Phylogenetic analysis of ITS, LSU, *RPB2*, and *TEF1* sequences revealed that *Distoseptispora daanyuanensis* exhibits a close phylogenetic relationship with *D. aquatica*, *D. nanchangensis* and *D. nanpingensis*. The nucleotide differences in the TEF1 and ITS regions between *Distoseptispora daanyuanensis* and *D.nanchangensis* are 15/815 (98.1% similarity) and 8/506 (98.4% similarity), respectively. The nucleotide differences in the *TEF1* and ITS regions between *Distoseptispora daanyuanensis* and *D.nanchangensis* are 17/815 and 6/506, respectively. Morphologically, differences exist in the conidiophores and conidia of *Distoseptispora aquatica*, *D. nanchangensis* and *D. nanpingensis*. The conidiophores of *Distoseptispora daanyuanensis* (25.5–46.7 × 1.3–2.4 μm; $\bar{x}$ = 38.4 × 1.8 μm, n = 20) are finer than those of *D. aquatica* (29–41 × 7–9 μm; $\bar{x}$ = 35 × 8 μm, n = 10) [1], *D. nanchangensis* (18.2–76.4 × 5.5–8.0 μm; $\bar{x}$ = 40.6 × 7.2 μm, n = 20) [40], and *D. nanpingensis* (8.5–28 × 5–7 μm; $\bar{x}$ = 18.1 × 6.1 μm, n = 10) [28]. Furthermore, the conidiophores of *Distoseptispora daanyuanensis* are characterized by being mycelium-like, while those of *Distoseptispora aquatica*, *D. nanchangensis* and *D. nanpingensis* are cylindrical. The conidia of *Distoseptispora daanyuanensis* have 4–92-distoseptate, whereas *D. aquatica*, *D. nanchangensis*, and *D. nanpingensis* are characterized by 15–28, (17–)21–43, and 28–41-distoseptate, respectively [14,28,40]. Consequently, *D. daanyuanensis* is recognized as a novel species within the genus *Distoseptispora*, supported by phylogenomic analyses and morphological characterizations.

**Figure 8:**
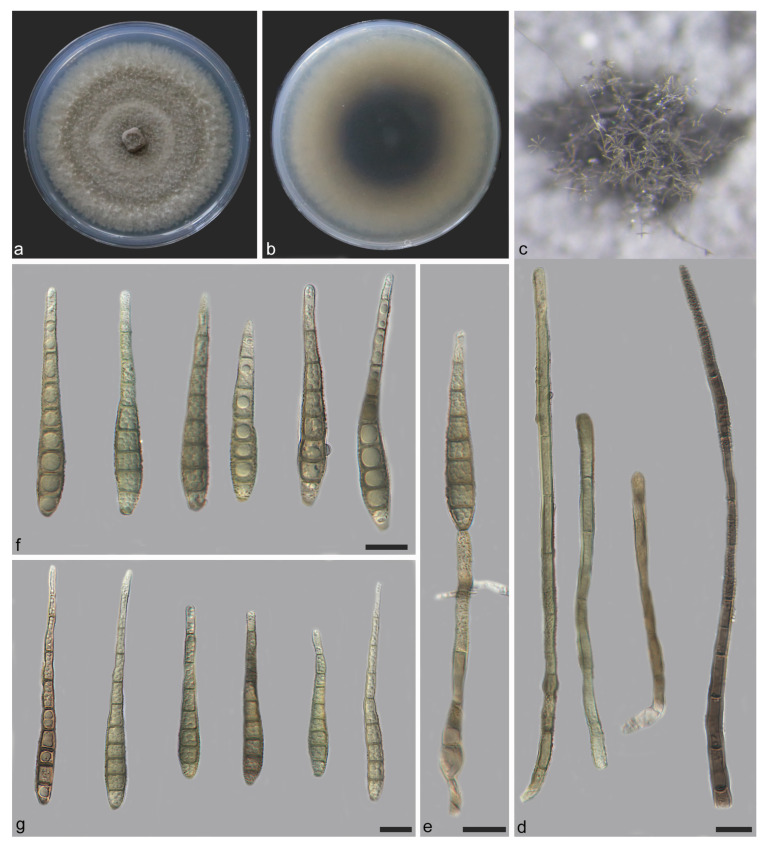
*Distoseptispora jianfenglingensis* (holotype: HMAS 354059). (**a**) surface of colony after 3 weeks on PDA; (**b**) reverse of colony after 3 weeks on PDA; (**c**) colonies on the substrate; (**d**) conidiophores; (**e**) conidiophores, conidiogenous cells and conidia; (**f**,**g**) conidia. Scale bars: (**d**–**g**) 10 μm.

**Figure 9:**
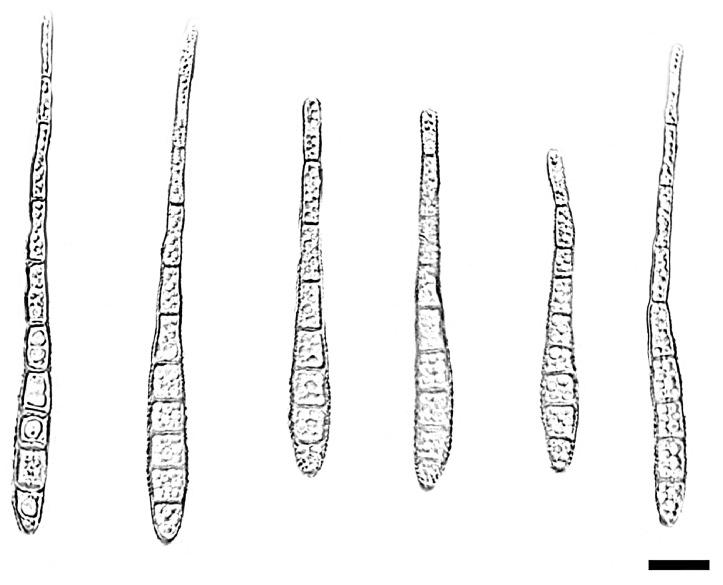
*Distoseptispora jianfenglingensis* (holotype: HMAS 354059). conidia. Scale bars: 10 μm.

*Distoseptispora jianfenglingensis* W.W. Liu, C.Z. Yin, X.G. Zhang & S. Wang, sp. nov. Figure 8 and Figure 9

MycoBank—MB859428

Holotype—China, Hainan Province, Ledong Li Autonomous County, Jianfengling National Nature Reserve (108°52′35″ N, 18°42′35″ E), from decaying wood, 12 April 2023, W.W. Liu, holotype HMAS 354059, ex-holotype living culture SAUCC WZS65-3.

Etymology—The epithet “*jianfenglingensis*” denotes the collection site of the strains, namely, Jianfengling National Nature Reserve.

Description—Saprobic on decaying wood in peat swamp forest. Sexual morph: Undetermined. Asexual morph: Hyphomycetous. Mycelium immersed to substratum, pale brown to subhyaline, smooth, septate, branched. Conidiophores macronematous, unibranchiate, cylindrical or rod-shaped, unbranched, upright, straight to slightly flexuous, smooth or micro-verrucose, dark brown, single, thick-walled, 4–13-septate with lobed basal cells, enteroblastic percurrent extensions, 66.5–164.2 × 3.8–5.7 μm ($\bar{x}$ = 119.8 × 4.2 μm, SD = 7.2 × 0.6, n = 20). Conidiogenous cells terminal, dark brown, integrated, monoblastic, holoblastic, cylindrical, smooth, 3.1–7.7 × 2.7–5.3 μm ($\bar{x}$ = 4.9 × 4.2 μm, SD = 1.9 × 0.8, n = 20). Conidia acrogenous, solitary, straight or slightly curved, brown to pale brown, rostrate, 5–10-distoseptate and most 9-distoseptate, swollen at the proximal end, tapering and paler toward the rounded apex, 43.3–76.1 × 5.1–7.5 μm ($\bar{x}$ = 53.0 × 6.5 μm, SD = 3.1 × 0.7, n = 25).

Culture characteristics—After 21 days of dark cultivation at 25 °C on PDA, the colony diameter reached 90 mm, and a growth rate of is 2.8–4.6 mm/day. Colonies circular, dense aerial mycelia, velutinous edge, dull surface, concentric, dark brown at the central parts, brown inner ring, outer pale brown halo, felted, dense, reverse concentric, blackish-brown inner ring, outer light brown halo.

Additional material studied—China, Hainan Province, Ledong Li Autonomous County, Jianfengling National Nature Reserve, from decaying wood, 12 April 2023, W.W. Liu, living culture SAUCC WZS65-4.

Notes—Phylogenetic analysis of ITS, LSU, *RPB2* and *TEF1* sequences revealed that *Distoseptispora jianfenglingensis* formed a well-supported monophyletic group with *D. liupanshuiensis*, indicating their close evolutionary relationship. The nucleotide differences in the *TEF1* and ITS regions between *Distoseptispora jianfenglingensis* and *D. liupanshuiensis* are 12/815 (98.5% similarity) and 5/506 (99% similarity), respectively. *Distoseptispora liupanshuiensis* is similar to *D. jianfenglingensis* but can be differentiated by having longer conidiophores (*D. liupanshuiensis*: 70–340 × 3.5–7 μm ($\bar{x}$ = 179 × 7.8 μm, n = 30) vs. *D. jianfenglingensis*: 66.5–164.2 × 3.8–5.7 μm ($\bar{x}$ = 119.8 × 4.2 μm, n = 20)); longer and thicker conidia (*D. liupanshuiensis*: 55–90 × 6–11 μm ($\bar{x}$ = 74 × 8.8 μm, n = 30) vs. *D. jianfenglingensis*: 43.3–76.1 × 5.1–7.5 μm ($\bar{x}$ = 53.0 × 6.5 μm, n = 25)); and differences in conidial septation (*D. jianfenglingensis*: 5–10-distoseptate vs. *D. liupanshuiensis*: 8–10-distoseptate) [38]. Consequently, *D. jianfenglingensis* is recognized as a novel species within the genus *Distoseptispora*, supported by phylogenomic analyses and morphological characterizations.

## 4. Discussion

*Distoseptispora* belongs to dematiaceous hyphomycetes and has high morphological similarity with the genera *Sporidesmium* Link and *Ellisembia* Subram [41]. *Distoseptispora*, like *Sporidesmium* and *Ellisembia*, possesses conidiophores, conidiogenous cells, and conidia, and the morphology of their conidia is very similar [42,43]. The conidia of *Distoseptispora* are darker in color and do not have a transparent attachment at the top, while the top of *Sporidesmium* sometimes has hyaline rounded apices [1]. Therefore, traditional taxonomy is no longer capable of distinguishing the “*sporidesmium*-like genera” from others [28]. In systematics, *Distoseptispora* is distinguished from *Sporidesmium* and *Ellisembia*, and it forms a distinct and well-supported clade sister to *Aquapteridospora* Jiao Yang, K.D. Hyde & Maharachch [39,44]. Therefore, *Distoseptispora* and *Aquapteridospora* are classified as two distinct genera within the order Distoseptisporales [45].

This study surveyed the fungal resources in two southern Chinese provinces, where the isolated *Distoseptispora* strains primarily originated from mountainous areas and nature reserves at altitudes ranging from 19.12 to 117.14 m.a.s.l. These strains have been found in both Hainan Province and Fujian Province, China, with Hainan Province containing the largest number, followed by Fujian Province. Our initial inference is that the mountainous areas and nature reserves in southern China, with their abundant vegetation and warm, humid climate, may harbor rich populations of *Distoseptispora* and other fungi. The plant specimens collected in this survey were decaying wood and decaying leaves, with all *Distoseptispora* samples obtained from the former and none detected in the latter. Therefore, except for endophytes, we infer that *Distoseptispora* has relatively weak host specificity for leaves and primarily parasitizes decaying wood.

*Distoseptispora*, a dematiaceous hyphomycete, has recently become a subject of taxonomic interest. Currently, we identify new species using morphological comparisons and multi-locus phylogenetic analyses (ITS, LSU, RPB2, and TEF1) with maximum-likelihood (ML) and Bayesian inference (BI) methods. A total of 94 records encompassing various species of Distoseptispora were retrieved from Index Fungorum (https://www.indexfungorum.org/, accessed on 6 May 2025), while 115 records, including ITS, LSU, RPB2, and TEF1 locus sequences, were recorded in the National Center for Biotechnology Information (NCBI) (https://www.ncbi.nlm.nih.gov/, accessed on 6 May 2025) and are compiled in the Supplementary Materials. This approach was additionally employed to characterize four novel species (*Distoseptispor bawanglingensis*, *D. changjiangensis*, *D. daanyuanensis* and *D. jianfenglingensis*) through comparative analyses. In this study, the *Distoseptispora* strain was cultivated on PDA medium, and colony characteristics were observed and recorded at 7, 14, and 21 days to enable macroscopic and microscopic examination and identification of the colonies. We found that, compared with other previously studied fungi, *Distoseptispora* grows very slowly on PDA medium. Specifically, the growth rate of *Distoseptispora daanyuanensis* is 0.9–1.6 mm per day, while that of *Daldinia menghaiensis* on PDA medium averages 12.8 mm per day [46], and the growth rate of *Beltraniella jianfengensis* is 9.2–10.2 mm per day [47]. Therefore, we will continue to optimize the culture medium suitable for the growth of *Distoseptispora* to accelerate the growth rate of the strain, as well as to discover and explore more species of this genus.

The genus *Distoseptispora* exhibits a widespread global distribution, as evidenced by data retrieved from the GlobalFungi database (https://globalfungi.com/; accessed: 5 May 2025), which encompasses 1144 specimens. Specifically, *Distoseptispora* was detected in Asia (90.03%), Africa (2.53%), South America (2.27%), North America (2.1%), Europe (1.84%) and Australia (1.22%). Of these, Asia exhibits a significantly larger sample size compared to other continents, encompassing 1030 samples. Over the past few years, a considerable number of novel species of *Distoseptispora* have been discovered, particularly in Thailand and China [41,48]. Samples analyzed in this investigation were sourced from Fujian and Hainan Provinces, which exhibit distinct climatic regimes: Subtropical Monsoon Climate and Tropical Rainforest Climate, respectively. These areas exhibit high levels of rainfall, persistent humidity, a high diversity of plant communities, and a significant fungal biodiversity, with *Distoseptispora* being one of the representative genera. Based on preliminary ecological assessments, we hypothesize that undisturbed montane ecosystems and old-growth forests, characterized by persistent mesic climatic regimes and luxuriant phytomass accumulation, may exhibit elevated species richness of *Distoseptispora* along with associated mycobiota. This proposition is supported by empirical evidence suggesting that such bioclimatic conditions facilitate fungal diversification through sustained resource availability and microhabitat complexity. In addition, *Distoseptispora* are primarily saprophytes found on woody substrates from freshwater habitats, some also have been found in terrestrial habitats and leaves [9,35]; Five *Distoseptispora* species (*D. bambusae*, *D. clematidis*, *D. tectonae*, *D. thysanolaenae*, and *D. xishuangbannaensis*) demonstrate habitat versatility, documented in both aquatic and terrestrial ecosystems [22]; Four *Distoseptispora* species (*D. caricis*, *D. licualae*, *D. thailandica* and *D. xishuangbannaensis*) parasitize on the leaves [7,27,45]. Accordingly, a comprehensive analysis will be conducted, considering geographical location, climate type, host specificity, and other relevant factors, followed by continued targeted sampling to identify additional *Distoseptispora* species.

## 5. Conclusions

Numerous novel fungal species were isolated from extensive decaying wood samples collected across Fujian and Hainan Provinces, China. Through comprehensive phylogenetic and morphological evaluations, four novel taxa belonging to the genus *Distoseptispora* were characterized. The virulence mechanisms and host–pathogen interactions of these newly identified *Distoseptispora* species remain poorly understood, requiring comprehensive investigations. Based on these findings, we propose that substrate-specific sampling of decaying wood could improve the discovery and taxonomic documentation of novel *Distoseptispora* taxa.

## Figures and Tables

**Table 1:** Differences between four new species of *Distoseptispora* and their closely related species.

Species	Colonies	Conidiphores (μm)	Conida			Habitat	Locality	Host
			Size (μm)	Morphology	Septation			
*Distoseptispora bawanglingensis* vs. *D. sichuanensis*	raised, circular, velutinous edge, grayish-brown, pale edge, surface velvety, felted, dense, reverse concentric, dark brown inner ring, outer pale brown halo vs. effuse, dark brown to black, hairy.	$13.9–40.6\;\times \;3.5–6.7\;(\bar{x}$ = 22.5 × 5.2, n = 20) $\mathrm{vs}.\;15–25\;\times \;4–6\;(\bar{x}$ = 20 × 5, n = 5)	$20.2–206.7\;\times \;8.3–13.7\;(\bar{x}$ = 141.7 × 10.4, n = 25) $\mathrm{vs}.\;80–145\;\times \;6–17\;(\bar{x}$ = 114 × 12.8, n = 20)	acrogenous or obclavate, solitary, straight or slightly curved, brown to pale brown vs. solitary, obclavate, elongated, straight or slightly curved, truncate at the base, rounded at the apex and hyaline, straight or slightly curved	3–45 vs. 12–20	Peat swamp forest vs. terrestrial	China	decaying Wood vs. dead branches
*D*. *bawanglingensis* vs. *D. xinpingensis*	raised, circular, velutinous edge, grayish-brown, pale edge, surface velvety, felted, dense, reverse concentric, dark brown inner ring, outer pale brown halo vs. effuse, brown to dark brown, solitary or gregarious	$13.9–40.6\;\times \;3.5–6.7\;(\bar{x}$ = 22.5 × 5.2, n = 20) vs. (97–)105–149(–175) × 4–5 μm ($\bar{x}$ = 127 × 5 μm, n = 40)	$20.2–206.7\;\times \;8.3–13.7\;(\bar{x}$ = 141.7 × 10.4, n = 25) $\mathrm{vs}.\;(95–)107–139(–155)\;\times \;(7–)8–9(–10)\;\mu m\;(\bar{x}$ = 123 × 8 μm, n = 40)	acrogenous or obclavate, solitary, acrogenous, straight or slightly curved, brown to pale brown vs. acrogenous, solitary, obclavate, truncate at base, tapering towards the apex, straight or slightly curved, brown, smooth, thin-wall	3–45 vs. 8–12	Peat swamp forest vs. freshwater stream	China	decaying Wood
*D. changjiangensis* vs. *D. olivaceoviridis*	circular, velutinous edge, low convex, concentric, light gray inner ring, outer dark gray, grayish-brown at the central parts, surface felted, dense, reverse dark brown, outer grayish white halo vs. single or in groups, numerous, hairy, dark brown	$12.0–173.5\;\times \;4.3–6.5\;(\bar{x}$ = 132.1 × 5.1, n = 20) vs. 34–78 × 5–7	$27.2–160.8\;\times \;12.1–15.8\;(\bar{x}$ = 153.6 × 14.1, n = 25) $\mathrm{vs}.\;46–115\;\times \;8–12\;(\bar{x}$ = 80 × 9 μm, n = 15)	acrogenous or obclavate, solitary, straight or slightly curved, brown to pale brown vs. acrogenous or obclavate, solitary, straight or slightly curved, brown to pale brown	6–40 vs. 3–45	Peat swamp forest vs. unknown	China vs. Thailand	decaying wood vs. *Clerodendrum quadriloculare*
*D. daanyuanensis* vs. *D. aquatica*	circular, white, with gray exudates, velutinous edge, dull surface, felted, dense, reverse concentric, blackish-brown inner ring, outer grayish white halo vs. effuse, scattered, hairy, dark brown, gray or black	$25.5–46.7\;\times \;1.3–2.4\;(\bar{x}$ = 38.4 × 1.8, n = 20) $\mathrm{vs}.\;29–41\;\times \;7–9\;(\bar{x}$ = 35 × 8 μm, n = 10)	$23.1–696.2\;\times \;9.3–17.9\;(\bar{x}$ = 302.4 × 309 11.3, n = 25) vs. 29–41 × 7–9	acrogenous, obclavate, solitary, straight or slightly curved, brown to pale brown vs. acrogenous, solitary, dry, obclavate, elongated, straight or slightly curved, truncate at the base, smooth, dark brown with bluish, green to malachite green tinge, paler towards the apex, thick- walled	4–92 vs. 15–28	Peat swamp forest vs. aquatic	China	decaying wood
*D. daanyuanensis* vs. *D. nanchangensis*	circular, white, with gray exudates, velutinous edge, dull surface, felted, dense, reverse con-centric, blackish-brown inner ring, outer grayish white halo vs. effuse, scattered, dark brown to black, and hairy	$25.5–46.7\;\times \;1.3–2.4\;(\bar{x}$ = 38.4 × 1.8, n = 20) $\mathrm{vs}.\;18.2–76.4\;\times \;5.5–8.0\;µm\;(\bar{x}$ = 40.6 × 7.2, n = 20)	$23.1–696.2\;\times \;9.3–17.9\;(\bar{x}$ = 302.4 × 309 11.3, n = 25) $\mathrm{vs}.\;149.1–292.7\;\times \;10.9–17.8\;(\bar{x}$ = 203.5 ×15.4, n = 30)	acrogenous, obclavate, solitary, straight or slightly curved, brown to pale brown vs. acrogenous, solitary, obclavate, straight or curved, brown to dark brown, smooth, thick-walled	4–92 vs. (17–)21–43	Peat swamp forest vs. unknown	China	decaying wood vs. dead branches
*D. daanyuanensis* vs. *D. nanpingensis*	circular, white, with gray exudates, velutinous edge, dull surface, felted, dense, reverse con-centric, blackish-brown inner ring, outer grayish white halo vs. effuse, scattered, dark brown to black, hairy	$25.5–46.7\;\times \;1.3–2.4\;(\bar{x}$ = 38.4 × 1.8, n = 20) $\mathrm{vs}.\;8.5–28\;\times \;5–7\;(\bar{x}$ = 18.1 × 6.1, n = 10)	$23.1–696.2\;\times \;9.3–17.9\;(\bar{x}$ = 302.4 × 309 11.3, n = 25) $\mathrm{vs}.\;169–282\;\times \;12–17.5\;(\bar{x}$ = 227.1 × 14.8, n = 20)	acrogenous, obclavate, solitary, straight or slightly curved, brown to pale brown vs. solitary, acrogenous, dry, obclavate, straight or curved, sometimes with a swollen cell, reddish-brown and slightly paler towards the apex, sometimes constricted at the septa, smooth	4–92 vs. 28–41	Peat swamp forest vs. terrestrial	China	decaying wood vs. dead branches
*D*. *jianfenglingensis* vs. *D. liupanshuiensis*	circular, dense aerial mycelia, velutinous edge, dull surface, concentric, dark brown at the central parts, brown inner ring, outer pale brown halo, felted, dense, reverse concentric, blackish-brown inner ring, outer light brown halo. vs. effuse, brown to dark-brown, hairy	$3.1–7.7\;\times \;2.7–5.3\;(\bar{x}$ = 4.9 × 4, n = 20) $\mathrm{vs}.\;70–340\;\times \;3.5–7\;(\bar{x}$ = 179 × 7.8, n = 30)	$43.3–76.1\;\times \;5.1–7.5\;(\bar{x}$ = 53.0 × 6.5, n = 25) $\mathrm{vs}.\;55–90\;\times \;6–11\;(\bar{x}$ = 74 × 8.8, n = 30)	acrogenous, solitary, straight or slightly curved, brown to pale brown, rostrate vs. acrogenous, solitary, straight, obpyriform, thick walled, light brown below, hyaline towards apex, rounded at the apex, truncate at base, tapering towards the apex	5–10 vs. 8–10	Peat swamp forest vs. unknown	China	decaying wood vs. dead culms of bamboo

**Table 2:** PCR primers, sequences and reaction condition used in this study.

Loci	PCR Primers	Sequence (5′–3′)	PCR Cycles
ITS	ITS4	GGA AGT AAA AGT CGT AAC AAG G	(95 °C at 30 s, 55 °C at 30 s, 72 °C at 1 min) × 35 cycles
	ITS5	TCC TCC GCT TAT TGA TAT GC	
LSU	LR0R	GTA CCC GCT GAA CTT AAG C	(95 °C at 30 s, 55 °C at 50 s, 72 °C at 1 min) × 35 cycles
	LR5	TCC TGA GGG AAA CTT CG	
*RPB2*	fRPB2-5F	CAT CGA GAA GTT CGA GAA GG	(95 °C at 45 s, 57 °C at 50 s, 72 °C at 90 s) × 40 cycles
	fRPB2-7cR	GGA RGT ACC AGT SAT CAT GTT	
*TEF1*	TEF1-983F	GCY CCY GGH CAY CGT GAY TTY AT	(94 °C at 30 s, 55 °C at 50 s, 72 °C at 1 min) × 35 cycles
	TEF1-2218R	AT GAC ACC RAC RGC RAC RGT YTG	

## Data Availability

The original contributions presented in this study are included in the article/supplementary material. Further inquiries can be directed to the corresponding author.

## Supplementary Materials

The following supporting information can be downloaded at https://www.mdpi.com/article/10.3390/jof11090667/s1. Table S1: The concatenated multiple sequences of ITS, LSU, RPB2, and TEF1. Table S2: Estimates of Evolutionary Divergence between Sequences [27].
