# Supplementary material for: Discovery and Identification of Four Novel Species of Distoseptispora (Distoseptisporaceae, Distoseptisporales) on Decaying Wood from Hainan and Fujian Provinces, China
Source: J Fungi (Basel). 2025 Sep 11;11(9):667. doi: 10.3390/jof11090667 (PMC12470301; doi:10.3390/jof11090667)
Supplement: Supplementary file 1 [file jof-11-00667-s001.zip › jof-3797300GenBank.pdf]

Table S1. GenBank numbers used in the phylogenetic analysis of *Distoseptispora*.

Taxon	Strain Number	GenBank Accession Numbers			
		ITS	LSU	*RPB2*	*TEF1*
Aquapteridospora fusiformis	MFLUCC 18–1606*	MK828652	MK849798	N/A	MN194056
A. lignicola	MFLUCC 15–0377*	MZ868774	KU221018	MZ892986	MZ892980
Distoseptispora adscendens	HKUCC 10820	N/A	DQ408561	DQ435092	N/A
D. amniculi	MFLUCC 17–2129*	MZ868770	MZ868761	MZ892982	N/A
D. appendiculata	MFLUCC 18–0259*	MN163009	MN163023	N/A	MN174866
D. aqualignicola	KUNCC 21–10729*	OK341186	ON400845	OP413474	OP413480
D. aquamyces	KUNCC 21–10732*	OK341187	OK341199	OP413476	OP413482
D. aquatica	MFLUCC 18–0646	MK828648	MK849793	N/A	MN194052
D. aquisubtropica	GZCC 22–0075*	ON527933	ON527941	ON533685	ON533677
D. arecacearum	MFLUCC 23–0212	OR354399	OR510860	OR481048	OR481045
D. atroviridis	GZCC 20–0511*	MZ868772	MZ868763	MZ892984	MZ892978
D. atroviridis	GZCC 19–0531	MW133915	MZ227223	N/A	MZ206155
D. bambusae	MFLUCC 20–0091*	MT232713	MT232718	MT232881	MT232880
D. bambusicola	GZCC 21–0667*	MZ474873	MZ474872	N/A	OM272845
D. bangkokensis	MFLUCC 18–0262*	MZ518205	MZ518206	N/A	OK067246
D. bawanglingensis	**SAUCC WZS13-1***	**PQ799295**	**PQ804721**	**PQ849357**	**PQ849363**
D. bawanglingensis	**SAUCC WZS13-2**	**PQ799296**	**PQ804722**	**PQ849358**	**PQ849364**
D. cangshanensis	MFLUCC 16–0970*	MG979754	MG979761	N/A	MG988419
D. caricis	CPC 36498*	MN562124	MN567632	MN556805	N/A
D. changjiangensis	**SAUCC WZS14-1***	**PQ799297**	**PQ804723**	**PQ849359**	**PQ849366**
D. changjiangensis	**SAUCC WZS14-1**	**PQ799298**	**PQ804724**	**PQ849360**	**PQ849365**
D. chinensis	GZCC 21–	MZ474871	MZ474867	N/A	MZ501609

	0665*				
D. chishuiensis	GZCC 23-0729*	PP584670	PP584767	N/A	PP663310
D. clematidis	MFLUCC 17–2145*	MT310661	MT214617	MT394721	N/A
D. clematidis	HJAUP C1319	PQ211102	PQ211110	PQ303676	PQ303681
D. crassispora	KUMCC 21–10726*	OK310698	OK341196	OP413473	OP413479
D. curvularia	KUMCC 21–10725*	OK310697	OK341195	OP413472	OP413478
D. cylindricospora	DLUCC 1906*	OK491122	OK513523	N/A	OK524220
D. daanyuanensis	**SAUCC 12326-1***	**PV670056**	**PV670405**	**N/A**	**PV708057**
D. daanyuanensis	**SAUCC 12326-2**	**PV670057**	**PV670406**	**N/A**	**PV708058**
D. dehongensis	KUMCC 18–0090*	MK085061	MK079662	N/A	MK087659
D. dipterocarpi	MFLUCC 22–0104*	OP600053	OP600052	OP595140	N/A
D. effusa	GZCC 19–0532*	MW133916	MZ227224	N/A	MZ206156
D. eleiodoxae	MFLUCC 23–0214	OR354398	OR510859	OR481047	OR481044
D. euseptata	MFLUCC 20–0154*	MW081539	MW081544	MW151860	N/A
D. euseptata	DLUCC S2024	MW081540	MW081545	MW084996	MW084994
D. fasciculata	KUMCC 19–0081*	MW286501	MW287775	N/A	MW396656
D. fluminicola	DLUCC 0391	MG979755	MG979762	N/A	MG988420
D. fluminicola	DLUCC 0999	MG979756	MG979763	N/A	MG988421
D. fujianensis	HJAUP C2509*	PQ211095	PQ211103	PQ303679	PQ303682
D. fujianensis	HJAUP C2513	PQ211098	PQ211106	PQ303680	PQ303683
D. fusiformis	GZCC 20–0512*	MZ868773	MZ868764	MZ892985	MZ892979
D. ganzhouensis	HJAUP C1090*	PQ211100	PQ211108	N/A	PQ303687
D. gasaensis	HJAUP C2034*	OQ942896	OQ942891	N/A	OQ944455
D. guanshanensis	HJAUP C1063*	OQ942894	OQ942898	OQ944458	OQ944452
D. guizhouensis	GZCC 21–0666*	MZ474868	MZ474869	MZ501611	MZ501610
D. guttulata	MFLU 17–0852*	MF077543	MF077554	N/A	MF135651

D. hainanensis	GZCC 22-2047*	OR427328	OR438894	OR449119	OR449122
D. hyalina	MFLUCC 17–2128*	MZ868769	MZ868760	MZ892981	MZ892976
D. hydei	MFLUCC 20-0481*	MT734661	MT742830	N/A	N/A
D. jianfenglingensis	**SAUCC WZS65-3***	**PQ799299**	**PQ804725**	**PQ849361**	**PQ849367**
D. jianfenglingensis	**SAUCC WZS65-4**	**PQ799300**	**PQ804726**	**PQ849362**	**PQ849367**
D. jinghongensis	HJAUP C2120*	OQ942897	OQ942893	N/A	OQ944456
D. lancangjiangensis	DLUCC 1864*	MW723055	MW879522	MW882260	N/A
D. lanceolatispora	GZCC 22-2045*	OR427329	OR438895	OR449120	OR449123
D. leonensis	HKUCC 10822	N/A	DQ408566	DQ435089	N/A
D. licualae	MFLUCC 14–1163A*	ON650686	ON650675	N/A	ON734007
D. licualae	MFLUCC 14–1163B*	ON650687	ON650676	N/A	ON734008
D. lignicola	GZCC 19-0529	MW133911	MZ227219	N/A	MZ206152
D. lignicola	HFJAU 0705 *	MK828651	MK849797	N/A	N/A
D. liupanshuiensis	GZCC 23-0730*	PP584669	PP584766	N/A	PP663309
D. longispora	HFJAU 0705*	MH555359	MH555357	N/A	N/A
D. longnanensis	HJAUP C1040*	OQ942887	OQ942886	N/A	OQ944451
D. martinii	CGMCC 3.18651*	KU999975	KX033566	N/A	N/A
D. meilingensis	JAUCC 4727*	OK562390	OK562396	N/A	OK562408
D. meilingensis	JAUCC 4728*	OK562391	OK562397	N/A	OK562409
D. menghaiensis	HJAUP C2045*	OQ942890	OQ942900	N/A	N/A
D. menghaiensis	HJAUP C2170	OQ942899	OQ942888	OQ944461	OQ944457
D. mengsongensis	HJAUP C2126*	OP787876	OP787874	N/A	OP961937
D. muchuanensis	CGMCC 3.27444	PQ067919	PQ067750	N/A	PQ278571
D. multiseptata	MFLUCC 15–0609*	KX710145	KX710140	N/A	MF135659
D. multiseptata	MFLU 17–0856	MF077544	MF077555	MF135644	MF135652
D. nabanheensis	HJAUP C2003*	OP787873	OP787877	N/A	OP961935

D. nanchangensis	HJAUP C1074*	OQ942889	OQ942895	OQ944460	OQ944454
D. nanpingensis	HJAUP C2517*	PQ211096	PQ211104	PQ303678	N/A
D. narathiwatensis	MFLUCC 23–0216	OR354400	OR510861	OR481049	OR481046
D. neorostrata	MFLUCC 18–0376*	MN163008	MN163017	N/A	N/A
D. nonrostrata	KUNCC 21–10730*	OK310699	OK341198	OP413475	OP413481
D. obclavata	MFLUCC 18–0329*	MN163012	MN163010	N/A	N/A
D. obpyriformis	MFLUCC 17–1694*	N/A	MG979764	MG988415	MG988422
D. obpyriformis	DLUCC 0867	MG979757	MG979765	MG988416	MG988423
D. olivaceoviridis	MFLU 24-0290	PQ568144	PQ569325	N/A	N/A
D. pachyconidia	KUMCC 21–10724*	OK310696	OK341194	OP413471	OP413477
D. palmarum	MFLUCC 18–1446*	MK085062	MK079663	MK087670	MK087660
D. phangngaensis	MFLUCC 16–0857*	MF077545	MF077556	N/A	MF135653
D. phragmiticola	GUCC 220201*	OP749887	OP749880	OP752699	OP749891
D. phragmiticola	GUCC 220202*	OP749888	OP749881	OP752700	OP749892
D. rayongensis	MFLUCC 18–0415*	MH457172	MH457137	MH463255	MH463253
D. rayongensis	MFLUCC 18–0417	MH457173	MH457138	MH463256	MH463254
D. rostrata	MFLUCC 16–0969*	MG979758	MG979766	MG988417	MG988424
D. rostrata	DLUCC 0885	MG979759	MG979767	N/A	MG988425
D. saprophytica	MFLUCC 18–1238*	MW286506	MW287780	MW504069	MW396651
D. septata	GZCC 22–0078*	ON527939	ON527947	ON533690	ON533683
D. sinensis	HJAUP C2044*	OP787878	OP787875	N/A	OP961936
D. sichuanensis	KUNCC 23-15519*	PP584672	PP584769	N/A	PP663312
D. songkhlaensis	MFLUCC 18–1234*	MW286482	MW287755	N/A	MW396642
D. suae	CGMCC 3.24262*	OQ874968	OQ732679	OQ870341	OR367670
D. submersa	MFLUCC 16-0946*	MG979760	MG979768	MG988418	MG988426
D. subtropica	HJAUP	PQ211099	PQ211107	PQ303677	PQ303684

	C2528*				
D. subtropica	HJAUP C2535	PQ211097	PQ211105	N/A	PQ303685
D. suoluoensis	MFLUCC 17–0224*	MF077546	MF077557	N/A	MF135654
D. suoluoensis	MFLUCC 17–0854	MF077547	MF077558	MZ945510	N/A
D. tectonae	MFLUCC 12–0291*	KX751711	KX751713	KX751708	KX751710
D. tectonae	MFLU 20–0262	MT232714	MT232719	N/A	N/A
D. tectonigena	MFLUCC 12–0292*	KX751712	KX751714	KX751709	N/A
D. thailandica	MFLUCC 16–0270*	MH275060	MH260292	N/A	MH412767
D. thysanolaenae	KUN–HKAS 112710	MW723057	MW879524	N/A	MW729783
D. thysanolaenae	KUN–HKAS 102247*	MK045851	MK064091	N/A	MK086031
D. tropica	GZCC 22–0076*	ON527935	ON527943	ON533687	ON533679
D. verrucosa	GZCC 20–0434*	MZ868771	MZ868762	MZ892983	MZ892977
D. wuzhishanensis	GZCC 22–0077*	ON527938	ON527946	N/A	ON533682
D. xinpingensis	KUNCC 22–12667*	OQ874970	OQ732681	OQ870340	OR367671
D. xishuangbannaensis	KUMCC 17–0290*	MH275061	MH260293	MH412754	MH412768
D. yichunensis	HJAUP C1065*	OQ942885	OQ942892	OQ944459	OQ944453
D. yongxiuensis	JAUCC 4725*	OK562388	OK562394	N/A	OK562406
D. yunjushanensis	JAUCC 4724*	OK562392	OK562398	N/A	OK562410
D. yunjushanensis	HJAUP C1307	PQ211101	PQ211109	PQ303675	PQ303686
D. yunnanensis	MFLUCC 20–0153*	MW081541	MW081546	MW151861	MW084995

Notes：New species established in this study are shown in bold. The ex-type strain are indicated using “*”. N/A: Not available.
